# The kinome, cyclins and cyclin-dependent kinases of pituitary adenomas, a look into the gene expression profile among tumors from different lineages

**DOI:** 10.1186/s12920-022-01206-y

**Published:** 2022-03-08

**Authors:** Keiko Taniguchi-Ponciano, Lesly A. Portocarrero-Ortiz, Gerardo Guinto, Sergio Moreno-Jimenez, Erick Gomez-Apo, Laura Chavez-Macias, Eduardo Peña-Martínez, Gloria Silva-Román, Sandra Vela-Patiño, Jesús Ordoñez-García, Sergio Andonegui-Elguera, Aldo Ferreira-Hermosillo, Claudia Ramirez-Renteria, Etual Espinosa-Cardenas, Ernesto Sosa, Ana Laura Espinosa-de-los-Monteros, Latife Salame-Khouri, Carolina Perez, Blas Lopez-Felix, Guadalupe Vargas-Ortega, Baldomero Gonzalez-Virla, Marcos Lisbona-Buzali, Daniel Marrero-Rodríguez, Moisés Mercado

**Affiliations:** 1 CONACyT-Unidad de Investigación Médica en Enfermedades Endocrinas, Hospital de Especialidades, Centro Médico Nacional Siglo XXI, Instituto Mexicano del Seguro Social, Av. Cuauhtémoc 330, Col. Doctores, D.F. 06720 Mexico, Mexico; 2 Instituto Nacional de Neurología Y Neurocirugía “Manuel Velasco Suarez”, Mexico, Mexico; 3 Centro Neurológico, Centro Medico ABC, Mexico, Mexico; 4 Área de Neuropatología, Servicio de Anatomía Patológica, Hospital General de México Dr. Eduardo Liceaga, Mexico, Mexico; 5 Facultad de Medicina, Universidad Nacional Autonoma de México, Mexico, Mexico; 6 Servicio de Endocrinologia, Hospital de Especialidades, Centro Medico Nacional Siglo XXI, Instituto Mexicano del Seguro Social, Mexico, Mexico; 7 Servicio de Neurocirugia, Hospital de Especialidades, Centro Medico Nacional Siglo XXI, Instituto Mexicano del Seguro Social, Mexico, Mexico

**Keywords:** Pituitary adenoma, Kinome, Cyclin, Cyclin-dependent kinase, Cell cycle, Pituitary tumors, Kinase

## Abstract

**Background:**

Pituitary adenomas (PA) are the second most common intracranial tumors and are classified according to hormone they produce, and the transcription factors they express. The majority of PA occur sporadically, and their molecular pathogenesis is incompletely understood.

**Methods:**

Here we performed transcriptome and proteome analysis of tumors derived from POU1F1 (GH-, TSH-, and PRL-tumors, N = 16), NR5A1 (gonadotropes and null cells adenomas, n = 17) and TBX19 (ACTH-tumors, n = 6) lineages as well as from silent ACTH-tumors (n = 3) to determine expression of kinases, cyclins, CDKs and CDK inhibitors.

**Results:**

The expression profiles of genes encoding kinases were distinctive for each of the three PA lineage: NR5A1-derived tumors showed upregulation of ETNK2 and PIK3C2G and alterations in MAPK, ErbB and RAS signaling, POU1F1-derived adenomas showed upregulation of PIP5K1B and NEK10 and alterations in phosphatidylinositol, insulin and phospholipase D signaling pathways and TBX19-derived adenomas showed upregulation of MERTK and STK17B and alterations in VEGFA-VEGFR, EGF-EGFR and Insulin signaling pathways. In contrast, the expression of the different genes encoding cyclins, CDK and CDK inhibitors among NR5A1-, POU1F1- and TBX19-adenomas showed only subtle differences. CDK9 and CDK18 were upregulated in NR5A1-adenomas, whereas CDK4 and CDK7 were upregulated in POUF1-adenomas.

**Conclusions:**

The kinome of PA clusters these lesions into three distinct groups according to the transcription factor that drives their terminal differentiation. And these complexes could be harnessed as molecular therapy targets.

**Supplementary Information:**

The online version contains supplementary material available at 10.1186/s12920-022-01206-y.

## Background

Pituitary adenomas (PA) are benign intracranial neoplasms that represent ~ 8% of all central nervous system tumors. The prevalence of these tumors ranges between 14–22% by autopsy and radiological studies in the general population [1]. PA are classified as either clinically functioning or non-functioning (CNFPA) depending on whether or not they result in a hormonal hypersecretion syndrome [2]. Clinically functioning PA comprise POU1F1-lineage derived tumors (GH-secreting somatotrophinomas, PRL-secreting prolactinomas and the rare TSH-secreting thyrotrophinomas) and the TBX19 lineage derived, ACTH-secreting corticotrophinomas. Most CNFPA are of gonadotrophic differentiation as they immunostain for α-subunit, LHβ and/or FSHβ and are conditioned by the transcription factor NR5A1 but also include silent corticotroph, somatotroph or lactotroph adenomas as well as null cell adenomas, which do not immunostain for any known hormone [3, 4].

Protein kinases are important for cellular signal transduction by regulating reversible phosphorylation events that play essential roles [5] and regulate key processes such as cellular proliferation, survival and migration, hence, they are well poised to contribute to several hallmarks of cancer if dysregulated [6]. Several kinases have been proven to be involved in pituitary tumorigenesis. For instance, STAT3 (signal transducer and activators of transcription type 3) has been associated with invasiveness in null cell adenomas [7], and to the up-regulation of GH hormone synthesis in somatotrophinomas [8]. Also, FGFR4 has been related to macroadenomas and with proliferation of pituitary adenomas [9]. Finally, abnormalities of kinases of the mTOR (mammalian target of rapamycin) pathway have also been linked to pituitary tumor development [10]. Several alterations in kinases signaling pathways lead to cell cycle proliferation [6]. Cell cycle is a highly regulated process that ensures duplication of genetic material and cell division [11]. This process is driven by several protein, among them cyclins and cyclin-dependent kinases (CDKs) and CDK inhibitors among others [12]. Interestingly, disruption of *CDKN1B*, the gene encoding the CDK inhibitor p27, along with overexpression of the gene encoding cyclin E, result in the development of pituitary tumor of the corticotroph [13], and cyclin D1 is up regulated in aggressive non-functioning tumor [14]. Down regulation of *CDKN2A*, the gene encoding the CDK inhibitor p16 and upregulation of cyclin D1 have been observed in a considerable proportion of aggressive CNFPA [14].

Although specific abnormalities in cell cycle regulation have been identified in several tumors, little is known regarding their potential role in pituitary gland development and pituitary tumor biology. Therefore, in the present study we looked into the expression profile of the different genes encoding kinases, cyclins, CDK as well as CDK inhibitors using a global transcriptomic and proteomic approach.

## Materials and methods

### Patients and tissue samples

Pituitary tumor samples were obtained at the time of surgery from 42 patients who were followed at the neuroendocrinological clinics of Hospital de Especialidades, Centro Médico Nacional Siglo XXI in Mexico City. Of these 42 patients, 20 had CNFPA, 10 had acromegaly, 6 had Cushing’s disease, 4 had TSH-secreting tumors and 2 had PRL-secreting macroadenomas. All patients, except those with prolactinomas were treatment naïve. Patients with prolactinomas had been previously treated with cabergoline but were considered to be resistant to the dopamine agonist, which was discontinued at least 3 months prior to surgery. Immunohistochemical characterization of the tumors was carried out as previously described [15]. Six non-tumoral pituitary glands were obtained within 10 h of death from autopsies performed at the Pathology Department of Hospital General de México and were used as controls. All participating patients were recruited with signed informed consent and ethical approval from the Comisión Nacional de Ética e Investigación Científica del Instituto Mexicano del Seguro Social in accordance with the Helsinki declaration [15].

### RNA purification

Total RNA was extracted from PA and non-tumoral pituitaries using the miRNAeasy Mini Kit (Qiagen Inc, CA, USA) according to manufacturer’s instructions. Tissue samples were disrupted and homogenized in 700 μl Qiazol Lysis Reagent. They were then incubated at room temperature for 5 min. Next, 200 μl of chloroform was added, and samples were incubated at room temperature for 3 min. The mixture was centrifuged at 12,500 rpm for 15 min at 4 °C. The aqueous phase was transferred to a fresh tube and mixed with an equal volume of 70% ethanol. Samples were then transferred to an RNAeasy Column in a 2 ml tube, and centrifuged at 10,000 rpm for 15 s. After centrifugation, 700 μl of RW1 buffer was added and the mixture was centrifuged at 10 000 rpm for 15 s. Flow-through was discarded and 500 μl of RPE buffer was added to the membrane and then centrifuged at 10 000 rpm for 15 s (2x). The column was transferred to a new collection tube adding 30 μl of RNAse free water and centrifuged for 1 min at 10 000 rpm. RNA was quantified using a Nanodrop-ND-1000 spectrophotometer (Thermo Scientific, DE, USA); RNA integrity was evaluated by Bioanalyzer 2100 [15].

### Microarray GeneChip Clariom D assay

The microarray used for these studies was Affymetrix Clariom D which allows us to analyze whole coding transcriptome at the gene and exon level as well as non-coding RNA such as lincRNA, miRNA and circRNA. Sample amplification and preparation for microarray hybridization was performed according to Affymetrix specifications. Briefly, 100 ng of total RNA was reversely transcribed into cDNA, amplified by in vitro transcription and reversely transcribed to cDNA again. Fragments between 40 and 70 bp were generated enzymatically, labelled and hybridized onto the microarray chips in an Affymetrix hybridization oven at 60 rpms and 45 °C for 17 h. Chips were washed according to the stablished protocols (Affymetrix, Santa Clara, CA, USA) with a GeneChip fluidics station 450, and finally scanned with an Affymetrix 7G GeneChip scanner. The raw data (CEL files) has been uploaded into the Gene Expression Omnibus (GEO), which is hosted by the National Center for Biotechnology Information (NCBI) under the accession number GSE147786 [15].

### Bioinformatic analysis of PA transcriptome

A total of 6 control and 42 PA experiments were analyzed, and two technical replicates. Data sets were analyzed by means of CEL files with the Expression Console, Partek Genomics Suite 7.19v software (Partek Incorporated, Saint Louis, MO, USA) and the Transcriptome Analysis Console (Affymetrix, Santa Clara, CA, USA). Pearson and Spearman correlations were performed, and probe sets were summarized by means of Median Polish and normalized by quantiles with no probe sets excluded from the analysis. Background noise correction was achieved by means of Robust Multi-chip Average (RMA) and data were log^2^ transformed. Data grouping and categorization was achieved by principal PCA. Differentially expressed genes were determined by means of ANOVA. Gene expression was considered to be altered upon identifying a + 2 or − 2 or + 1.5 or − 1.5 fold change compared to non-tumoral pituitaries, *p* ≤ 0.05 and FDR ≤ 0.05 parameters [15].

### Pathway, enrichment networks, protein–protein interactions

Enrichr (https://maayanlab.cloud/Enrichr/) and Metascape (http://metascape.org/gp/index.html#/main/step1) was used for understanding the biological meaning behind the resulting list of genes, to obtain gene ontology and pathway information for significantly de-regulated genes in pituitary lesions. The enrichment networks were carried out using Metascape. Protein–Protein Interactions (PPI) identification was carried out using Metascape.

### Protein purification

The Plasma Membrane Protein Extraction Kit (Abcam) was used according to manufacturer’s specifications. Briefly, tissues were washed in ice-cold phosphate buffered saline (PBS) 1X as many times were needed to eliminate most of the blood present. The tissue was homogenized in 2 mL of ice-cold Homogeneize Buffer with Halt Protease and Phosphatase Inhibitor Cocktail 1X (Thermo) in a BeadBug Microtube homogenizer (Benchmark). The homogenate was centrifuged at 700 g during 10 min at 4 °C to collect tissue and cells that were not lysed. The supernatant was transferred to a new tube and was centrifuged at 10,000 *g* during 30 min at 4 °C. Pellet formed correspond to all membrane proteins and supernatant correspond to cytosolic proteins. Cytosolic proteins were precipitated with four volumes of 95% acetone and centrifuged [16].

### Sample preparation for proteomic analyses

The proteins were dissolved in 20 μL of 0.2% Protease Max Surfactant (Promega) in 50 mM NH4HCO3 and 15 μL of UREA 8 M (Sigma-Aldrich). The equivalent to 200 μg of protein was reduced with 10 mM dithiothreitol (Sigma-Aldrich) at 37 °C for 60 min and alkylated with 20 mM iodoacetamide (Sigma-Aldrich), for 30 min at room temperature under the dark, then Tris–HCl pH 8.6 (Promega) was added to reach 10 mM. Digestion was made with trypsin (Promega) 1:35 at 37 °C overnight and then peptides were fractionated with HyperSep SCX cartridges (Thermo Scientific) following the manufacturer's instructions. Five fractions were obtained from each sample which were desalted with Sep-Pak tC18 cartridges (Waters), dried in a SpeedVac concentrator (Eppendorf), and kept at − 80 °C. The samples were reconstituted in 30 µl of 0.1% formic acid and 5% acetonitrile, centrifuged at 20,000 g at 4 °C for 5 min and injected on a C18 Nano HPLC column for separation of peptides [16].

### Nano-HPLC–MS/MS analysis

The peptide solutions (5 µl) were loaded into a Dionex UltiMate 3000 HPLC system (Thermo Scientific) using a pre-column/peptide trap Acclaim PepMap 100 C18 (300 µm × 1.5 cm) (Dionex), and a separation column Acclaim PepMap RSLC C18 (75 µm × 15 cm) (Dionex). Chromatographic runs were performed at a constant flow of 300 nL/min of a mixture of 0.1% (v/v) formic acid in water (Buffer A, from a Milli-Q system), and 0.1% (v/v) formic acid in acetonitrile (Buffer B, HPLC grade from Sigma-Aldrich) in a linear gradient of 85 min from 2–40% B. At min 90, the gradient increased to 90% B and was held there for 11 min after which the percentage of B was returned to 2% for column re-equilibration. Electrospray ionization of the eluted peptides was performed with a CaptiveSpray source (Bruker) assisted by a flow of nitrogen boiled on acetonitrile (0.2 bar) and the mass spectra were acquired with a quadrupole time-of-flight mass spectrometer (Impact II, Bruker). Positive ions were analyzed over an m/z range of 100–2200. Before every six injections, calibration was performed with the ESI-TOF Tuning mix (Agilent). MS/MS fragmentation was performed for those ions with a signal higher than 5000 counts applying a cycle time of 3 s and excluding + 1 charged ions. Active exclusion was active after one spectrum for 2 min, unless the intensity of the precursor was more than three times higher than in the previous scan. Collision energy depended on the precursor ion charge and mass (e.g. at 700 m/z, 33 eV and 27 eV for 2 + and 3 + ions respectively; whereas at 1100 m/z, 65 eV and 55 eV were used for 2 + and 3 + ions) [16].

### Database searching and analysis of proteomic data

Protein identifications were made processing the raw files with the DataAnalysis-otof-default script from the Bruker Compass DataAnalysis software (version 4.4 SR1, Bruker), the Protein Scape software (version 3.1.3 461, Bruker) using Mascot 2.4.1 (Matrix Science): trypsin as the digestion enzyme, two missed cleavages allowed, carbamidomethyl Cys as a fixed modification and oxidation on Met as variable modification. Monoisotopic peptide masses were searched with 7.0 ppm peptide mass tolerance and 0.05 Da fragment mass tolerance. FDR was set to 1% with the peptide decoy and percolator options active. The SwissProt database for Homo sapiens was used. Proteins with Mascot scores > 13 were considered as successful identifications [16].

### mRNA and protein correlation

Venn diagrams was performed to correlate the mRNA and protein expression using http://bioinformatics.psb.ugent.be/webtools/Venn/.

## Results

We have recently described that transcriptome and proteome of pituitary adenomas segregate into three distinct clusters, according to the transcription factor that drives their terminal differentiation: NR5A1-derived gonadotrophinomas which constitute the majority of CNFPA; TBX19-derived clinically evident ACTH-secreting tumors causing Cushing disease; and POU1F1-derived somatotrophinomas, prolactinomas and thyrotrophinomas [15, 16].

### Kinase gene expression profile in pituitary tumors

**Fig. 1:**
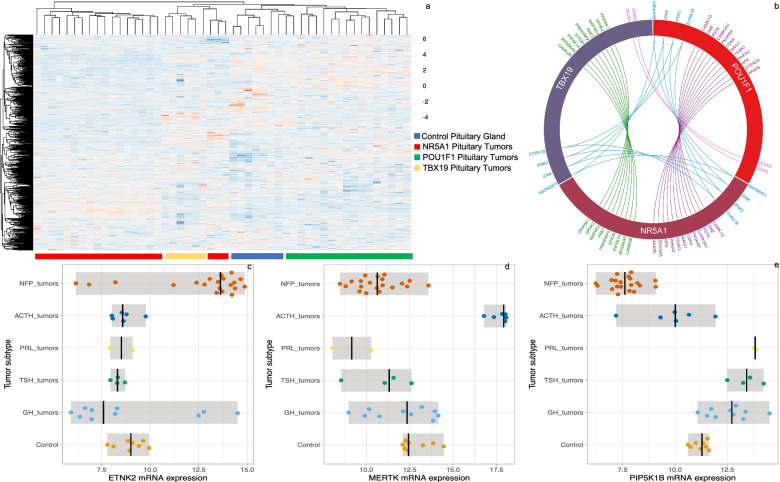
Kinase expression profile in pituitary tumors. Panel **a** depicts the hierarchical cluster from kinase expression profile segregating the three tumor lineages. Panel **b** circos plot of the kinase shared between tumor lineages, purple lines and letter depicts kinases shared by NR5A1 and POU1F1 tumor lineages, green lines and letters depicts kinases shared between NR5A1 and TBX19 tumor lineages, blue lines and letters depics kinases shared between the three tumor lineages and magenta lines and letter depicts the kinases shared between POU1F1 and TBX19 tumor lineages. Panel **c**, **d**, and **e** showed the expression of ETNK2, MERTK and PIP5K1B in NR5A1-, TBX19- and POU1F1 tumor lineages, respectively

**Fig. 2:**
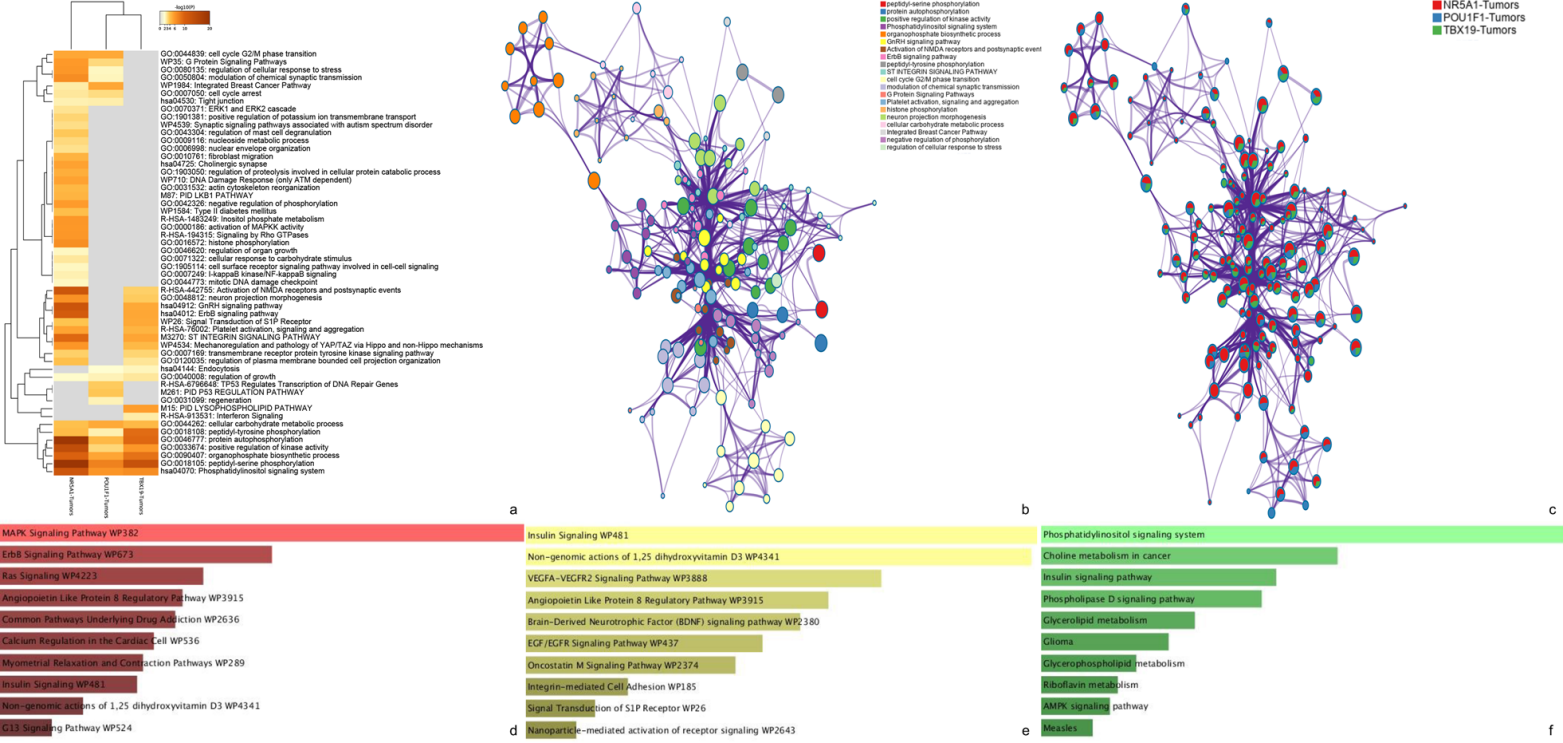
Gene ontology, pathway and network analysis. Panel **a** shows hierarchical cluster of the deregulated pathways in each tumor lineage. Panel **b** and **c** displays network analysis and each tumor lineage input to each node, respectively. Panels **d**, **e** and **f** displays gene ontology from expressed kinases in NR5A1, TBX19 and POU1F1 tumor lineages, respectively

The kinase profile segregates the pituitary tumors according to their late transcription factor driving pituitary cytodifferentiation, and the gene ontology (GO) analysis indicate that there are divergent and distinctive events in each tumor lineage (Figs. 1 and 2).

Kinases such as ETNK2 (*p* = 0.0001) and PIK3C2G (*p* = 0.0009) characterize NR5A1 lineage tumors, PIP5K1B (*p* = 3.63e−05) and NEK10 (*p* = 0.0005) distinguish POU1F1 lineage and MERTK (*p* = 5.05e−08) and STK17B (*p* = 1.04e−08) describe TBX19 lineage (Fig. 1). The three tumor lineages share only four kinases and have a large proportion of lineage-specific kinases. The shared kinases in all tumors included IP6K2, MAPK8IP3, IDNK and DYRK1B. POU1F1- and NR5A1-tumors shared genes such as CAMK1G, GNE, PSTK and CAMK2N1 among the twelve shared kinases. NR5A1- and TBX19-tumors shared CAMK2B, RPS6KA5, PIP5K1A and STK26 among the nine shared kinases. And finally, POU1F1- and TBX19-tumors shared two kinases, DCLK3 and DGKG (Fig. 1).

The NR5A1 kinome showed kinases related to MAPK, ErbB and RAS signaling, whereas POU1F1 kinase profile showed phosphatidylinositol, insulin and phospholipase D signaling pathways and TBX19 lineage displayed VEGFA-VEGFR, EGF-EGFR and Insulin signaling pathways (Fig. 2). Interestingly, Mechanoregulation and pathology of YAP/TAZ (Yes-associated protein/WWcontaining transcriptional regulation protein 1) via Hippo and non-Hippo mechanisms were altered only in NR5A1 and TBX19 tumors. Attractively, the three tumor lineages showed alteration in Phosphatidylinositol (PI) signaling system.

These results indicate that pituitary tumors from the three different lineages prefer characteristic and divergent signal transduction pathways that could represent the basis for specific molecular targeted therapy for each tumor lineage.

Network analysis of the kinases up regulated in the three tumor lineages showed interaction between several nodes such as ErbB, positive regulation of kinase activity, peptidyl-serine phosphorylation and phosphatidylinositol signaling system, in the biggest and central node. This central node interacts with cell cycle G2/M phase transition node and histone phosphorylation node (Fig. 2).

Several of the genes encoding kinase presented isoforms, resulting from alternative splicing of mRNA: kinases PAK7 and STK26 in NR5A1-derived tumors, kinase PPIP5K2 in POU1F-derived adenomas and kinases like PPIP5K2 and kinases WEE1 and STK17 in the TBX19 tumors.

### NR5A1-derived pituitary adenomas cyclins and cyclin-dependent kinases

**Fig. 3:**
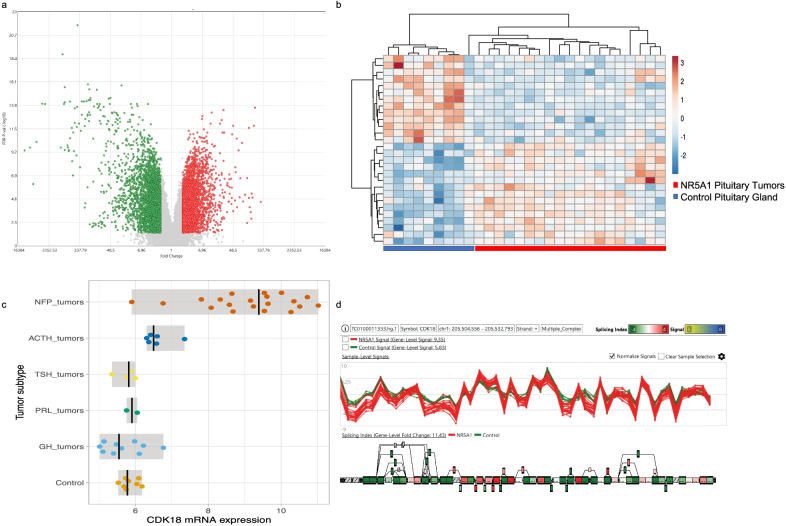
Transcriptome analysis from NR5A1-derived tumors. Panel **a** shows volcano plot of the differentially expressed genes in CNFPA from gonadotrope, null cell and silent ACTH tumors. Panel **b** hierarchical cluster from the differentially expressed cyclins and CDK in CNFPA tumors. Panel **c** depicts the CDK18 up-regulation in CNFPA and panel **d** the potential mRNA isoforms that could be present in these tumors

The clinically non-functioning pituitary adenomas exhibited the most differentially expressed cyclins and cyclin-dependent kinases. Genes encoding CDK18 and CDK9 were significantly up-regulated (*p* = 4.32E−12 and *p* = 2.29E−09, respectively). Genes encoding cyclin-G1 (CCNG1), cyclin-D1 (CCND1) and cyclin-E2 (CCNE2) were also found to be up-regulated (*p* = 2.7E−06, *p* = 0.0025 and *p* = 0.0005, respectively). Remarkably, the majority of down-regulated genes were those encoding CDK inhibitors such as CDKN1A (p21cip1) (*p* = 3.79E−09), CDKN2A (p16ink4a) (*p* = 7.98E−12) and CDKN2C (p18ink4c) (*p* = 5.70E−09) as well as cyclins such as Cyclin-J-like (CCNJL) (*p* = 0.0002), Cyclin-A2 (CCNA2) (*p* = 1.97E−06), Cyclin-D3 (CCND3) (*p* = 0.0001), Cyclin-H (CCNH) (*p* = 1.50E−07) and CDK2 (*p* = 0.0007) (Fig. 3).

Besides being upregulated in CNFPA, genes encoding CDK18, Cyclin-G2 and Cyclin-D1 also presented alternative splicing isoforms in PA (Fig. 3).

### POU1F1-derived pituitary adenomas cyclins and cyclin-dependent kinases

**Fig. 4:**
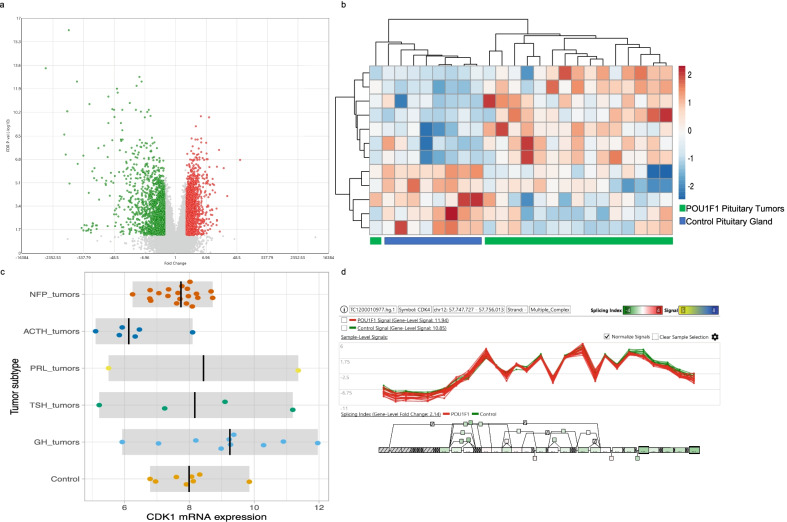
Transcriptome analysis from POU1F1-derived tumors. Panel **a** shows volcano plot of the differentially expressed genes in POU1F1 from GH-, TSH- and PRL-secreting tumors. Panel **b** hierarchical cluster from the differentially expressed cyclins and CDK in POU1F1 tumors. Panel **c** depicts the CDK1 up-regulation in POU1F1 and panel **d** the lack of mRNA isoforms these in tumors

The up regulated genes were those encoding CDK4 (*p* = 0.0018), CDK7 (*p* = 0.0002) and Cyclin-K (CCNK) (*p* = 7.61E−05), whereas the down regulated genes were those encoding Cyclin-JL (CCNJL) (*p* = 0.0006), Cyclin-D1 (CCND1) (*p* = 0.0005), CDK2 (*p* = 0.0002) and the CDK inhibitor CDKN2A (p16^ink4a^) (*p* = 0.0001) (Fig. 2). mRNA alternative splicing was not found in any of these differentially expressed genes which may suggest that POU1F1-derived adenomas require these CDK and cyclins in their intact form (Fig. 4).

### TBX19-derived adenomas cyclins and cyclin-dependent kinases

**Fig. 5:**
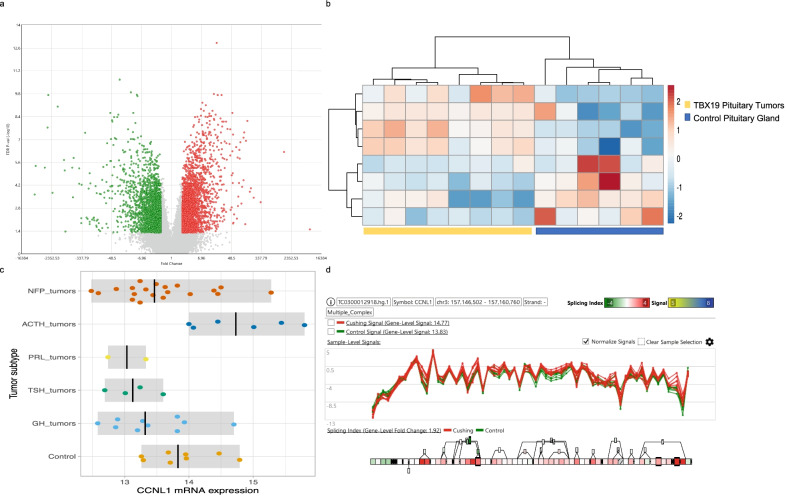
Transcriptome analysis from TBX19-derived tumors. Panel **a** shows volcano plot of the differentially expressed genes in TBX19 from ACTH-secreting tumors. Panel **b** hierarchical cluster from the differentially expressed cyclins and CDK in ACTH secreting tumors. Panel **c** depicts the CCNL1 expression in TBX19 tumors and panel **d** the potential mRNA isoforms that could be present in these tumors

TBX19-derived tumors, which consist of ACTH-secreting adenomas, had the lowest number of differentially expressed cyclin, CDK and CDK inhibitor genes. Genes encoding CCNL1 (*p* = 0.0071), Cyclin-B2 (CCNB2) (*p* = 0.0001) and Cyclin-F (CCNF) (*p* = 0.0013) were up regulated, whereas genes encoding CDK inhibitors CDKN1B (p27^kip1^) (*p* = 0.0012) and CDKN2C (*p* = 1.75E−07) and cyclin Cyclin-JL (CCNJL) (*p* = 8.49E−05) were down-regulated. The vast majority of genes were equally expressed in tumor and non-tumoral, control pituitary glands, including CCNL1, CCNE2, CCNB2, CCNA1, CDK18, CDK19 and CDK20 (Fig. 5). Despite not being differentially expressed, genes encoding Cyclin-L1 (CCNL1) and Cyclin-F (CCNF), as well as CDK19 and CDK17 presented potential mRNA isoforms (Fig. 5).

### Looking for a pituitary tumor subtype-specific cyclin, CDK and CDK-inhibitor expression profile

**Fig. 6:**
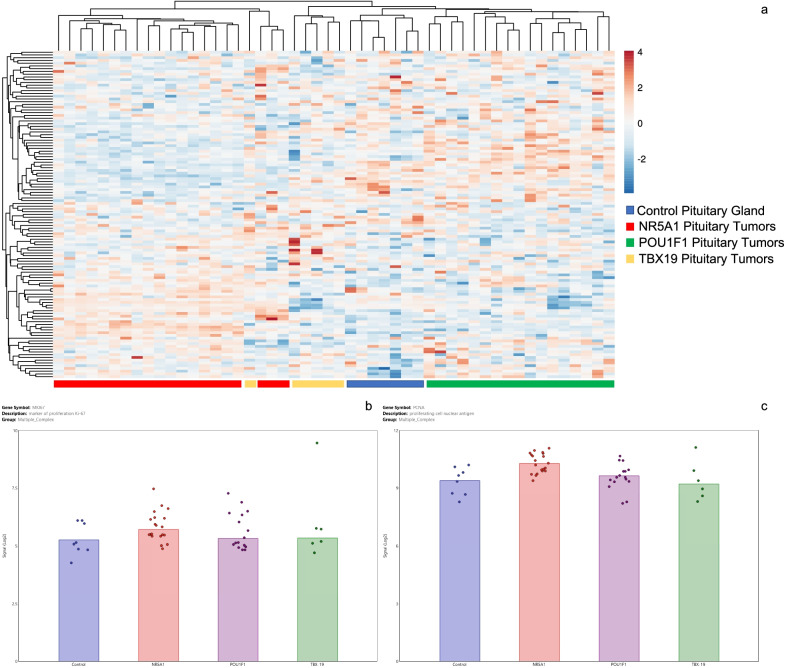
Cyclins and CDKs expression profile in all pituitary tumor subtypes. Panel **a** Heatmap depicting the hierarchical cluster from cyclin and CDKs mRNA expression in pituitary tumors derived from TBX19 (yellow), POU1F1 (green) and NR5A1 (red) tumors. Tumors clustered according to their transcription factor that determine tumor lineage. Panel **b** and **c** shows expression of KI67 and PCNA proliferation genes in pituitary tumor lineages

Although the expression of some genes seemed to be specific for certain adenoma subtypes, such as CDK18 in NR5A12-derived tumors and CDK1 in POU1F1-derived tumors, we did not find a profile that could exclusively characterize a particular tumor subtype. Thus, NR5A1-, POU1F1- and TBX19-derived adenomas shared the same expression level of most genes encoding the different cyclins, CDK and CDK inhibitors (Fig. 6). In concordance with this finding, the expression of other genes involved in the control of cell proliferation such as Ki-67 (*p* = 0.5438) and PCNA (Proliferating cell nuclear antigen *p* = 0.6637), was found to be the same among the different adenoma subtypes (Fig. 6).

### Pituitary tumors proliferation profiles

The G1 and G1/S transition gene expression profiles partially segregates the tumors according to their origin lineage or their late transcription factors driving pituitary cytodifferentiation, whereas G2 and G2/M transition gene expression profiles does not show any tumor nor lineage specific profile (Supplementary Fig. 1). Particularly, the G1 and G1/S transition expression profiles differentiates better the NR5A1 from the POU1F1 and TBX19 tumor lineages, supporting the observation of the cyclins and CDK expression where CNFPA present more proliferation than the other tumors. The potential finding that NR5A1 derived tumors proliferate more than POU1F1- and TBX19-derived tumors could be due to the fact that they do not present a clinical syndrome due to lack of hormone hyper secretion therefore they behave more silently until they present compression symptoms, whereas POU1F1 and TBX19 are readily detectable because the consequences of the hormones side effect. Although we could not match this finding with a high Ki67 and PCNA indices, these markers are generally very low in PA, reflecting their benign nature.

### Protein correlates with mRNA

For the correlation between transcriptomic and proteomic analysis, we performed Venn diagram showing several mRNA genes and proteins correlation. As is well known, there was no one hundred percent similarities between the two molecules. For the NR5A1-lineage tumors, besides some of the above mentioned in kinome and cyclin sections, we found CDK18 again, WNK2, PAK3 and STK33 among others. As for the POU1F1-lineage tumors CDK9 and CDK4 appears again, ADPGK and STRAP also showed correlation in mRNA and protein, again besides the mentioned in previous sections. Unfortunately, TBX19-lineage tumors were not analyzed by proteomic experiments because of their relatively small size sample.

## Discussion

Protein kinases are key enzymes that regulate a wide range of biological processes such as cellular proliferation, survival and migration [6]. Molecular abnormalities in one or several of these complex enzymes are at the forefront of oncogenesis. The term kinome alludes to the complete set of protein kinases encoded by the genome. The mitogen activated protein kinase (MAPK) signaling pathway is frequently involved in tumor initiation and progression, as well as in the development of resistance to chemotherapy [17]. This signaling pathway was significantly altered in NR5A1-derived CNFPA. The gene encoding ETNK2 was also found to be up regulated in our patients with CNFPA. This kinase is involved in cellular proliferation and resistance to apoptosis, as well as in tumor invasion and migration and is frequently overexpressed in breast and gastric malignancies [18, 19]. The EGFR is a tyrosine kinase receptor that is frequently upregulated in several cancers such as lung, colon, head and neck, pancreas and breast [20]. The gene encoding this receptor, was also found to be upregulated in TBX19 tumors, as was the gene encoding MERTK which seems to play an important role in tumor proliferation, survival and migration [21]. Finally, the POU1F1-derived tumors showed alterations in phosphatidylinositol signaling pathways which is altered in neuroendocrine tumors, hematopoietic malignancies, breast, colon and gastric cancer [22]. The gene encoding PIP5K1B, known to participate in the regulation of cell cycle, proliferation, migration and apoptosis [23] was significantly overexpressed in POU1F1 adenomas. Targeting kinases with oncogenic transformational capacity has led to notable changes in the management of cancer. Attractively, YAP/TAZ Hippo and non-Hippo were altered in in two lineages, NR5A1 and TBX19. These molecules have been previously related to poorly differentiated pituitary tumors and could have prognostic value and opening venues for new treatments [24]. Remarkably, the three tumor lineages showed Phosphatidylinositol signaling alterations and has been shown to be activated in pituitary tumors [25]. Currently, the Federal Drug Administration (FDA) has approved single and multiple kinase inactivators that target a limited number of enzymes. Kinase inhibitors are very efficacious in the treatment of several malignant tumor [26], and may be useful in the treatment of invasive pituitary adenomas and the very rare pituitary carcinomas.

Inherently kinases are related to cell cycle progression, which is driven by cyclins, they bind and activate the cyclin-dependent kinases. Specific heterodimeric cyclin–CDK complexes phosphorylate a plethora of cellular proteins to promote entry and progression of the cell cycle [27]. Cyclins D1, D2, and D3 activates CDK4-6 and facilitates progression during G1. CDK2/cyclin E complexes become active at the end of G1 and participate in the transition from G1 to S phase. At the end of the S phase and during G2, cyclin E is substituted by cyclins A1/A2 activating CDK2 and CDK1. Finally, CDK1/cyclin B (mostly B1 and B2) complex is involved in progression through G2 and entry into the M phase [28].

Molecular alterations in cell cycle regulation involving the disruption of cyclins, CDK and CDK inhibitors are common events in pituitary oncogenesis [29]. It has been estimated that approximately 80% of the pituitary tumors could harbor alterations in at least one of the cell cycle regulators [12].

Interestingly, in our results the NR5A1-derived CNFPA presented the highest number of differentially expressed genes encoding cyclins CDK and cyclin inhibitors. CDK18 is one of the least known CDKs and appears to play a role in signaling cascades of terminally differentiated cells and in the regulation of genome integrity [30]. Cyclins D1 and D3, which activate CDK4, are often overexpressed in PA, particularly in CNFPA [31], 32. Interestingly, genes encoding CDK inhibitors, such as CDKN1A (p21^Cip1^), CDKN2A (p16^INK4A^), CDKN2C (p18^INK4C^) were downregulated in our CNFPA, which is consistent with a cyclin-mediated oncogenic mechanism, whereby these proteins act as tumor suppressors. Deletion of p18INK4C in mice results in pituitary hyperplasia and adenoma formation [33]. Hypermethylation of the promoter region of the gene encoding p16^INK4A^ occurs in more than 70% of CNFPA, with the corresponding absence of protein [34]. Cyclin E levels are uniquely increased in corticotroph tumors but undetectable in normal pituitary [32]^34^. Roscovitin (seleciclib), a purine analog that inhibits the CDK/Cyclin E complex, has been shown to inhibit ACTH secretion by tumoral corticotrophs in vitro and is currently undergoing phase II trials in patients with Cushing disease [35]. Although in our transcriptomic study, neither Cyclin E nor CDK2 genes were found to be upregulated in TBX19-derived tumors, we did find a significant down regulation of the gene encoding their cognant inhibitor CDKN1B (p27^kip1^), as well as of CDKN2C (p18^INK4C^).

Proliferation markers such as KI67 and PCNA showed no differential expression between the three tumor lineages or between tumoral tissues and non-tumoral pituitaries. This is consistent with the slow growth rate of the pituitary tumors [36, 37].

Our results revealed that several cyclins that could potentially undergo alternative splicing in pituitary tumors. Alternative splicing is a ubiquitous regulatory mechanism of gene expression that allows generation of more than one unique mRNA species from a single gene resulting in formation of different protein isoforms. Approximately 90–95% of the genome undergoes alternative splicing which contributes to cell differentiation, lineage determination, tissue identity and organ maintenance and development [38]. Splicing in tumors can affect crucial genes related to processes such as proliferation, angiogenesis, apoptosis, as well as cellular energetics [39]. Cyclins such as CCNL1 experience alternative splicing generating several mRNA and protein isoforms, which has been shown to participate in mRNA translation and apoptosis [40, 41]. Likewise, CCND1 has been shown to generate mRNA and protein isoforms that could participate in tumorigenesis [42] Correspondingly, CDK2 mRNA isoforms can have an impacts on cell cycle [43]. The mechanisms of alternative splicing and the tumor-specific isoforms could be harnessed as therapy targets.

## Conclusion

We have shown that the kinase expression profile of pituitary adenomas clusters these lesions into three distinct groups according to the transcription factor that drives their terminal differentiation. The expression of certain cyclins, CDK and cyclin-inhibitor genes appears to be rather lineage-specific. Our findings open up the possibility of therapeutically targeting some of these enzymes in order to treat patients with large, invasive and recurrent pituitary adenomas.

## Supplementary Information

**Additional file 1. Supplementary figure 1.** Cell cycle stages gene expression in pituitary adenomas. Panel **A**) shows the G1 stage of the cell cycle gene expression profile in the three lineages of pituitary tumors and control gland. Panel **B**) display the G1/S transition gene expression profile, **C**) portray the G2 expression profile and **D**) the G2/M transition gene expression profile. Blue depicts the control gland, red the NR5A1 tumors, green the POU1F1 tumors and yellow the TBX19 tumors respectively.

## Data Availability

The raw data (CEL files) has been uploaded into the Gene Expression Omnibus (GEO), which is hosted by the National Center for Biotechnology Information (NCBI) under the accession number GSE147786.
