# Supplementary material for: The kinome, cyclins and cyclin-dependent kinases of pituitary adenomas, a look into the gene expression profile among tumors from different lineages
Source: BMC Med Genomics. 2022 Mar 8;15:52. doi: 10.1186/s12920-022-01206-y (PMC8905767; doi:10.1186/s12920-022-01206-y)
Supplement: Supplementary file 1 — Additional file 1. Supplementary figure 1. Cell cycle stages gene expression in pituitary adenomas. Panel A) shows the G1 stage of the cell cycle gene expression profile in the three lineages of pituitary tumors and control gland. Panel B) display the G1/S transition gene expression profile, C) portray the G2 expression profile and D) the G2/M transition gene expression profile. Blue depicts the control gland, red the NR5A1 tumors, green the POU1F1 tumors and yellow the TBX19 tumors respectively. [file 12920_2022_1206_MOESM1_ESM.pdf]

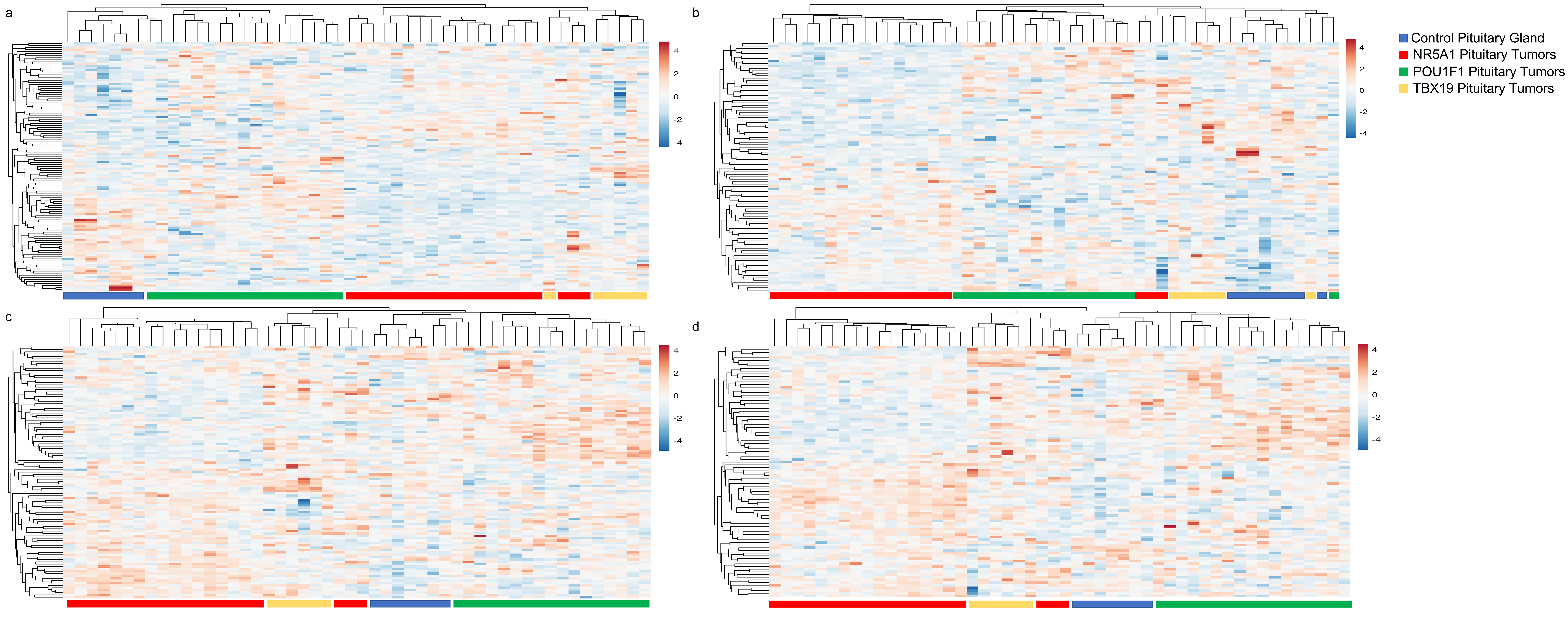

Supplementary figure 1.- Cell cycle stages gene expression in pituitary adenomas. Panel A) shows the G1 stage of the cell cycle gene expression profile in the three lineages of pituitary tumors and control gland. Panel B) display the G1/S transition gene expression profile, C) portray the G2 expression profile and D) the G2/M transition gene expression profile. Blue depicts the control gland, red the NR5A1 tumors, green the POU1F1 tumors and yellow the TBX19 tumors respectively.
